# Single shot of 17D vaccine may not confer life-long protection against yellow fever

**DOI:** 10.1590/0074-02760170347

**Published:** 2017-11-27

**Authors:** Pedro FC Vasconcelos

**Affiliations:** Instituto Evandro Chagas, Ananindeua, PA, Brasil

**Keywords:** yellow fever vaccine, single dose vaccine, multiple dose vaccine, WHO, life-long immunity

## Abstract

The yellow fever (YF) vaccine has been used since the 1930s to prevent YF, which is a severe infectious disease caused by the yellow fever virus (YFV), and mainly transmitted by Culicidae mosquitoes from the genera *Aedes* and *Haemagogus* . Until 2013, the World Health Organization (WHO) recommended the administration of a vaccine dose every ten years. A new recommendation of a single vaccine dose to confer life-long protection against YFV infection has since been established. Recent evidence published elsewhere suggests that at least a second dose is needed to fully protect against YF disease. Here, we discuss the feasibility of administering multiple doses, the necessity for a new and modern vaccine, and recommend that the WHO conveys a meeting to discuss YFV vaccination strategies for people living in or travelling to endemic areas.

Yellow fever (YF) is an infectious disease that is endemic in sub-Saharan Africa and tropical South America, and is transmitted by Culicidae mosquitoes of the genera *Aedes* , *Haemagogus* , and *Sabethes* . *Aedes aegypti* is the urban vector, which has been associated with large epidemics in the past on both sides of the Atlantic Ocean. In Africa, many species of *Aedes* are sylvatic vectors, including *A. africanus* , *A. simpsoni* , and others ( WHO 1985 ), while the main vectors in South America are *Haemagogus janthinomys* in northern and central regions and *H. leucocelaenus* in the Southern Cone, and *Sabethes* species are secondary vectors ( Monath & Vasconcelos, 2015 , Vasconcelos & Monath 2016) .

YF is a severe disease with high case fatality rate (CFR), especially in South America where the average CFR is 50% of reported cases, but ranges from 30-80% ( Monath & Vasconcelos 2015 ). Historically, the urban cycle has been responsible for the most severe epidemics observed in both endemic regions in previous centuries ( WHO 1985) .

The development and use of the 17D vaccine in the 1930s ( Theiler & Smith 1937 ) dramatically reduced the incidence of YF, and effectively stopped its transmission in urban settings in the New World. For decades, the World Health Organization (WHO) recommended vaccination every ten years for people including travellers and those living in endemic areas (WHO 1985, 2008, Monath 2001) .

The immunogenicity of the vaccine is lower in children ( Nascimento Silva et al. 2011 ). In an interesting serologic study using different assays, Niedrig et al. (1999) showed that 10 years after receiving yellow fever virus (YFV) vaccination around 25% of vaccines had no neutralisation antibodies, suggesting that a booster is necessary to maintain protective levels of neutralising antibodies.

The recent epidemics of YF in Angola and Brazil in 2016 and 2017, respectively, re-opened the question of the necessary number of doses of YFV required, because of the occurrence of YF in previously vaccinated people. In 2013, the WHO recommended a single dose of YFV to confer life-long protection against YF (WHO 2013). While the decision was taken unanimously, it was based on old studies; and Brazil chose not to adopt the recommendation following national discussions. However, this was revised by the Brazilian Ministry of Health in 2017 after the largest epidemic in the country since the urban cycle was eliminated in the 1940s, and the country has since temporarily adopted the single vaccine dose due a shortage in 17D vaccine supply.

The polemic of a single, double, or multiple YF vaccine doses over the lifespan of those in endemic areas is an open question to investigate, and the decision should be scientifically based and preferentially on recent data. In particular, there are logistical and technical challenges inherent in the production of the 17D vaccine. It is necessary to recapitulate some facts in light of the recent epidemics in the Old World (Angola and Democratic Republic of Congo) and New World (Brazil), which have occurred in the last two years:

- The initial WHO decision was based on the shortage of YF vaccine (WHO 2013);
- The shortage occurred because 17D vaccine production is limited, laborious, and empirical. The price per dose is low, and therefore there is no market-driven incentive to produce it;
- The shortage of 17D vaccine resulted in the use of fractional doses during the 2016 epidemic in Kinshasa city, the capital of the Democratic Republic of Congo, and evaluating the immunogenicity of this approach will take several years;
- The WHO stockpile of 6 million doses funded by the GAVI alliance is insufficient to guarantee a fast and efficient response to the global re-emergence of YF. The occurrence of several YF cases originating in China highlights this weakness;
- To increase the production of 17D vaccine it is necessary to improve and modernise production plants;
- The cost of plant modernisation is extremely high and is therefore not attractive to the WHO’s prequalified producers;
- It is therefore necessary to develop a new and modern YF vaccine that is economically attractive to the vaccine production industry, has increased safety against severe adverse viscerotropic disease, and is at least as immunogenic as 17D;
- It may be more than a decade before this modern vaccine is available, and depending on the approach used will require several doses to guarantee lifelong protection.

At least two recent studies in Brazil showed that the level of neutralising antibodies decreases dramatically in adults and children eight and four years after primary vaccination, respectively ( Caldas et al. 2014 , Campi-Azevedo et al. 2016 ). In addition, between 1980 and 2017, 29 cases of severe and frequently fatal sylvatic YF cases were reported in people previously vaccinated in Brazil, not including cases in the most recent epidemic ( PAHO 2016 ). This supports the recommendation of at least two vaccine doses to confer complete protection against YF. This is especially significant because of the re-emergence of YF in Brazil and other South American endemic countries ( Vasconcelos 2010 ). According to Niedrig et al. (1999) , at least 25% of individuals that received a single shot did not have neutralising antibodies after 10 years, suggesting that a second dose as a booster is needed to maintain protective levels of antibodies.

In light of this information, the recommendation of a single dose of 17D vaccine is not reasonable, and may result in deaths that could have been prevented with an additional vaccine dose. An alternative strategy that might overcome the vaccine shortage is reducing the quantity of virus per dose to around 1000 PFU/dose, because the amount in each vaccine dose is currently at least ten times more than the recommended amount.

The WHO urgently needs to convey a meeting with vaccine specialists, epidemiologists, and virologists, together with the WHO prequalified producers of the 17D vaccine to review this topic, and to stimulate the production and/or modernisation of the 17D vaccine.
